# The transition process between child and adolescent mental services and adult mental health services for patients with anorexia nervosa: a qualitative study of the parents’ experiences

**DOI:** 10.1186/s40337-021-00404-w

**Published:** 2021-04-13

**Authors:** Veronica Lockertsen, Lill Ann Wellhaven Holm, Liv Nilsen, Øyvind Rø, Linn May Burger, Jan Ivar Røssberg

**Affiliations:** 1 Division of Mental Health and Addiction, Oslo University Hospital, P.O. Box 4959, Nydalen, Oslo, Norway; 2 Institute of Clinical Medicine, Faculty of Medicine, University of Oslo, 0318 Oslo, Norway; 3 Oslo, Norway; 4 Regional Department for Eating Disorders, Division of Mental Health and Addiction, Oslo University Hospital, Ullevål HF, Postboks 4950 Nydalen, 0424 Oslo, Norway; 5 Drammen, Norway

**Keywords:** Anorexia nervosa, Mental health transition, Adolescent, Carers, Parents’ perspective, Caregivers’ perspective, Qualitative research, Service user’s perspective

## Abstract

**Background:**

Patients with Anorexia Nervosa (AN) often experience the transition between Child and Adolescent Mental Health Services (CAMHS) and Adult Mental Health Services (AMHS) as challenging. This period tends to have a negative influence on the continuity of care for the adolescents and represents a demanding and difficult period for the parents. To our knowledge, no previous study has explored the parents’ experience with the transition from CAMHS to AMHS. Therefore, this qualitative study examines how parents experience the transition process from CAMHS to AMHS.

**Methods:**

In collaboration with a service user with carer experience, qualitative interviews were conducted with 10 parents who had experienced the transition from CAMHS to AMHS, some from outpatient care and others from both in- and outpatient mental care units in Norway. All had some experience with specialized eating disorder units. The interviews were analyzed with a Systematic Text Condensation (STC) approach. Service users’ perspectives were involved in all steps of the research process.

**Results:**

Six categories represent the parents’ experiences of the transition: (1) the discharge when the child turns 18 years old is sudden; (2) the lack of continuity is often followed by deterioration and relapses in the patient; (3) the lack of involvement and information causes distress; (4) knowledge – an important factor for developing a trusting relationship between parents` and clinicians`; (5) parents have overwhelming multifaceted responsibilities; and (6) parents need professional support.

**Conclusion:**

Improving the transition by including parents and adolescents and preparing them for the transition period could ease parental caregiving distress and improve adolescents’ compliance with treatment. Clinicians should increase their focus on the important role of parents in the transition process. The system should implement routines and guidelines to offer caregivers support and guidance during the transition process.

## Plain English summary

Adolescents with anorexia nervosa (AN) who receive care from Child and Adolescent Mental Health Services (CAMHS) often need further treatment from Adult Mental Health Services (AHMS). This study explores the transition between CAMHS and AHMS for parents of adolescents with AN in a naturalistic public mental health-care setting. Our study identified six themes concerning the transition between CAMHS and AMHS, based on parents’ experiences of the process and what they considered influenced the transition. In general, parents and patients are both unprepared for the sudden discharge from CAMHS when the patient turns 18 years old. The transition is often characterized by a lack of continuity, leading to deterioration and relapses among patients. The lack of involvement and information parents experience in the transition causes distress, and parents have overwhelming multifaceted responsibilities in the transition period. Parents view knowledge to be the key to a successful transition, and they are stressed and need support in the transition.

## Introduction

Treatment guidelines recommend family interventions for adolescents with Anorexia Nervosa (AN) [1, 2]. Parents are often involved in the care while the adolescent AN patient is treated by Child and Adolescent Mental Health Services (CAMHS), but their role decreases when transitioning to Adult Mental Health Services (AMHS) [3]. The average onset for AN is the mid-teens, with an average duration of 6 years [4]. Thus, many patients are treated by both CAMHS and AMHS [5].

The development of AN is caused by a complex interplay of biological, psychological, social, developmental, and cultural factors. AN can manifest in a variety of ways, but often patients are ambivalent about treatment and do not regard themselves as ill [1, 6]. This ambivalence with treatment and fluctuating motivation for recovery creates additional challenges in the management of a successful transition.

Blum [7] defined an optimal transition as a purposeful, planned process that addresses the medical, psychosocial, and educational/vocational needs of adolescents. Many mental health-care systems fail to complete a seamless transition, mostly because of the lack of good cooperation between the services and different treatment philosophies [4, 8]. Four features of optimal transition have been identified: Information transfer, a period of parallel care, transition planning, and continuity of care [9]. Singh et. al studied transitions in an UK context involving six mental health trusts. They found that the majority of service users experience the transition as poorly planned, poorly executed, and poorly experienced [9, 10]. Studies show that only 4–13% of patients with a mental health disorder experience a satisfactory transition [11]. One of the most critical factors for a successful transition is preparation. Preparation implies acknowledging how important the therapeutic relationship in CAMHS can be in the adolescents’ lives, and how leaving a secure relationship influences how the new relationship is established in AMHS [12–15].

Parents have an important caregiving role in treating patients suffering from AN, but their role changes when entering AMHS. In CAMHS, parents are used to having responsibility for the patients when it comes to both meal support and weight restoration. However, AMHS places the responsibility on the patient [3]. The different treatment approaches often create a barrier in the transition, as both patient and parents are often unprepared, and the changes are not made explicit. When parents experience themselves as being less involved in the treatment of AMHS, it can increase their feelings of loss and fear and lead to a sense of powerlessness [4, 8, 16]. Patients with AN often have disabilities that make them rely on their parents for emotional and financial support, even though they are emerging into adulthood [17]. Therefore, the transition should be guided by an individual transition plan that considers the need for the individual to practice self-sufficiency and family involvement [9, 18].

The emotional impact of caring for an individual with mental health problems is well established (Baronet, 1999). Parents of patients with AN appear to have poorer mental wellbeing than parents of other conditions [19]. Kyriacou, Treasure [20] found that more than 50% of parents with AN scored above the clinical threshold for anxiety, and 13% scored above the clinical threshold for depression. To decrease their own distress and increase involvement in the treatment of their adolescent, the parents expressed a need for information and support from the services [21, 22]. Without support, there is an increased risk of developing maladaptive coping strategies in their interactions with adolescents. To avoid conflict, parents can often develop family routines that in fact contribute to the adolescent maintaining their AN symptoms [23, 24].

Parents play an essential role in the transition period. However, to our knowledge, no previous studies have explored the parents’ experiences of the transition from CAMHS to AMHS for patients with AN. Greater knowledge about how parents experience the transition process and the factors they experience as barriers to a more satisfactory transition for the patient might improve the transition process. This knowledge can also provide us with important information about what the parents need during the transition period to provide support for adolescents with AN. The main aim of this qualitative study was to examine how parents experience the transition process from CAMHS to AMHS.

## Methods

### Design

The present study is part of a service user-initiated qualitative research project focusing on the transition from CAMHS to AMHS for patients [14], professionals [24], and parents. The project is inspired by how the theme of challenging transitions to AMHS appeared repetitively in support groups for parents with children suffering from eating disorders. A parent with service use experience (LAWH) participated in the design of a semi-structured interview guide and moderated the interview in collaboration with the first author (VL). The research group also included a former service user (LMB) with patient experience, RN nurses (LN, VL), and psychiatrists (JIR and ØR).

### Recruitment and participants

The study recruited 12 participants, three fathers and nine mothers, through snowball sampling methods. Eight of the participants were individual parents and four were couples; thus, the transitions of 10 adolescents were discussed. Three of the participants were invited to participate by their adolescents’ therapists, who contacted participants they knew had experiences that could illuminate the research questions. The others made contact with the project manager after finding project information on an internet support site for eating disorders. None of the parents had relations to the patients included in a previous study [14]. The adolescents were aged 15, on average, when they first contacted the CAMHS. All parents had experience with the transition from CAMHS to AMHS. All but two of the adolescents had experience with in-patient treatment, and all had some experience with specialized eating disorder units. All parents had the main care of the adolescent while transitioning; one had received assistance from child welfare services until the transition. Three of the parents were separated and had the main responsibility for the adolescent during the transition. All transitions occurred the year their adolescents turned 18. The average time from transition to the qualitative interview was 4.5 years. Recruitment to the study took place between September 2018 and December 2019 in Norway. Written consent was obtained from all participants.

### Data collection

In-depth, semi-structured interviews, guided by a thematic interview guide, were conducted by the first (VL) and the second (LAWH) authors. The interviews lasted between 60 and 90 min and were conducted in adapted settings chosen by the participants. The interviews were guided by our research question, which was to explore parents’ experiences of parenting an adolescent with AN in the transition from CAMHS to AMHS. The participants were encouraged to describe their experience with the transition through specific examples. The questions focused on factors that influenced the transition process, regarding the treatment systems, the therapists, themselves, and their adolescents. Throughout the interview, we restated and summarized our understanding to validate our interpretations of their narratives. The first author audio-recorded and transcribed the interviews verbatim.

### Qualitative data analysis

Data were analyzed using a systematic text condensation (STC) inspired by Giorgi [25, 26]. STC is an elaboration of Giorgi’s principles, and the method focused on presenting the parents’ expressed experiences, rather than a possible underlying interpretation of meaning. STC is a descriptive approach, presenting the participants’ experience as expressed by themselves. However, STC shares the underlying theoretical foundations of social constructionism and has in this study been used more dynamically than the procedure may imply. By de-contextualization and re-contextualization of the text, we analytically reduced the data and created a condensed text that represented the overall material [25]. Using STC, the moderators of interviews were considered co-producers of the data, and together with the participants, affected the interview process. STC comprises four main steps, where the end goal is to have a condensed text representing the validity and wholeness of the original context. Three of the authors listened and read the interviews several times, focusing on the parents’ experiences with the transition between CAMHS and AMHS. Our research question developed dynamically as the research process influenced our understanding of the parents’ experiences. After forming an overall impression of the material, texts that belonged together were grouped and encoded. By decontextualizing selections of meaning content from the created codes, we were able to reveal various aspects of the parents’ experiences. This systematic way of renaming codes and processing the material was constantly conducted with the authenticity of the interviews in mind. Finally, our analysis revealed six themes that represented the answer to our research question. We used NVivo 11 to help organize and categorize the transcriptions (QSR International Pty Ltd.).

## Results

Six categories describe the parents’ experiences during the transition: (1) the discharge when the child turns 18 years old is sudden; (2) the lack of continuity is often followed by deterioration and relapses in the patient; (3) the lack of involvement and information causes distress for the parents; (4) parents have overwhelming multifaceted responsibilities in the transition; (5) knowledge is the key to a successful transition; and (6) parents are stressed and need support. The following paragraphs discuss each category in greater detail.

### Sudden discharge from CAMHS at 18

The parents described the transition from CAMHS to AMHS as an abrupt end to treatment that is defined by age rather than a process. The parents were told that AMHS was responsible for the treatment when the patients turned 18 years old and that a transition phase with parallel care was impossible. The parents experienced stress, as they were unsure of how to proceed in the treatment system when their son/daughter was suddenly discharged from CAMHS. The parents felt powerless, with the sense that they had nothing to say, and they described the feeling of being left out of what was happening with their adolescent in AMHS.

The worst thing about the transition process is how she was discharged from outpatient care on her 18th birthday without any form of follow-up. She was all alone, and we, as parents, were not included or notified.

The parents expressed concern about the transition from CAMHS that was processed without consideration of the adolescents’ capabilities or maturity.

I understand that the system must have limits, but she was treated like an adult from the first meeting between CAMHS and AMHS. When you are 18, you are not an adult. So, it should have been a transitional phase.

She met the CAMHS-therapist on Friday, turned 18 on Sunday—the following Monday she met with AMHS. She had been warned about how the transition was regulated by the calendar, but she was totally caught off guard.

One of the parents experienced that CAMHS wanted to continue treatment for a period, but the adolescent wanted to transfer to AMHS for a fresh start. To their surprise, CAMHS just let the adolescent go, without any contact information or help to transfer to AMHS.

She was all alone, and we, as parents, were not included or notified. There was a sharp separation between CAMHS and AMHS. CAMHS would not give us any assistance. They could at least have recommended someone or connected us with the outpatient clinic in AMHS.

### Lack of continuity is often followed by deterioration and relapses

The parents frequently described the transition as going in circles between services, hospitalizations, and meetings with their primary care provider (PCP). For the parents, the transition period implied having to wait for adequate treatment, handle unstable adolescents, and face uncertainties about future treatment. This situation was described as a negative circle, putting the responsibility of the adolescent’s health in the hands of the PCP and the parents and highlighting the important role continuity of treatment plays in the transition.

It is such a negative circle. Our PCP referred us to AMHS. It takes about 6 weeks to get an appointment at the outpatient clinic. Soon after the first meeting with our daughter, the therapist was going on vacation and she was left without a therapist. Soon after the vacation, the therapist went on paternity leave and our daughter was discharged without further consideration. Suddenly, once again, her PCP was her only therapist. This circle is not unique.

The parents described how their adolescents’ health often deteriorated in waiting periods, with some patients being admitted to an acute psychiatric ward. Both the waiting time and the admission to the acute ward were difficult for the parents. The waiting time represented a period of feeling frightened and solely responsible for their adolescent. However, they found that the acute psychiatric ward was not attuned to their adolescent’s needs and challenges, and they found it difficult leaving them in what they felt was an inhumane environment.

During the transition period, much of the parents’ time was spent searching for adequate treatment facilities. The criteria for receiving treatment from AMHS were considered strict and focused mostly on the adolescent’s BMI.

How thin must one become to receive treatment? Actually, she lost weight just to meet their BMI criteria.

The parents described how their adolescent’s ambivalence toward treatment created high levels of frustration during the transition period. The patients often dropped out of treatment, and the parents felt they were left responsible and followed up more closely as they were afraid of the consequences. How adolescents would become responsible for their own health without continuous professional support or preparation was of significant concern for the parents.

Although they considered it natural and important that their adolescents had something to say about treatment, they wanted the clinician to acknowledge the role patients’ ambivalence plays in treating adolescents with AN and the impact it has on their self-sufficiency. They had experienced that their adolescent’s disease made them make decisions they, in their parents’ eyes, should not take.

She refused potassium supplements because in her mind, she gained weight. That was more important than surviving. As she had turned 18, they gave her that choice and handed her a paper to sign, confirming she was familiar with the consequences of refusing treatment with a potentially fatal outcome.

### Lack of involvement and information causes parental distress

The parents experienced a change in their role as caregivers when their adolescents started receiving treatment from AMHS. Despite still being responsible for the adolescent, after transferring to AMHS, there was a difference in their inclusion in the treatment and in the amount of information they received. The parents described how the lack of information made life at home difficult. They described a need for more guidance and knowledge about how to interact with their son/daughter.

I understand that some things are confidential, but I literally got nothing and had to handle it on my own. With more information, I could have understood how serious it was, sooner.

Access to information was a topic already present in CAMHS, as the duty of confidentiality is set at age 16. Nevertheless, some describe a sudden change in their adolescents’ attitudes to accessing AMHS. The parents experienced that the emphasis of AMHS on the individual’s independence and self-sufficiency influenced the adolescents’ attitudes. Parents that earlier had good cooperation with their adolescent and thereby access to information suddenly lost their overview of the situation.

I felt, since it came so suddenly after transitioning, almost from 1 day to the next—it must have been the influence AMHS had. The idea of when you are 18, you are more responsible for your own life.

This sudden change in attitudes toward their involvement made the transition challenging for the parents. They often experienced that the amount of information they received and their cooperation with the adolescent were dependent on the adolescent’s somewhat fluctuating state of disease. An easier flow of information to and from parents could benefit the transition. They questioned why their experience and knowledge were not requested by the health-care services, as in their view, it would make it easier for the clinician to understand the adolescent. Often, the adolescents found it straining to connect with a new clinician; thus, a more involved parents’ role would be a positive bridging factor.

I tried to speak with the doctors, but they looked at me like I was a nuisance. And as we did not receive any information, we kind of lost a way to understand her, so we felt very insecure and scared. They did not even let us know when she had escaped from the unit!

### Knowledge – an important factor for developing a trusting relationship between parents and clinician’s

The parents described how a successful transition often relied on trust in the clinician’s competence with eating disorders. The adolescents tended to lose motivation for treatment and had difficulties in the therapeutic relationship when they sensed that the clinician knew less about eating disorders than they did themselves.

Developing trust was difficult, as N is really smart and has much knowledge about her own disease. She pulled him up on misinformation and then believed that he did not have the necessary competence.

During the transition, the parents experienced meetings with clinicians who seemingly lacked knowledge about what their caregiving role implied. Some parents described being met with a distrustful attitude, as they sensed that the professionals found it difficult to assess and trust their narrative. While some meetings in the transition increased their confidence and decreased their distress concerning their role as parents, others described how clinicians had increased their feelings of shame and lack of confidence in how they handled their situation. Consequently, their room for manoeuvre shrank during the transition period, and they felt lost.

There is no understanding of the parents’ situation. Everywhere else I have been has confirmed that my reaction was normal to an abnormal situation. But it ruined me.

When met by experienced clinicians who embraced their situation and recognized their reactions, they felt calmer and had greater acceptance of their reactions. Health services ignoring the parents’ knowledge and expertise in the transition was also of symbolic value to some parents. They used it as an example of what they perceived as professionals’ tendency to focus on the somatic symptoms of the eating disorder, while overlooking the multidetermined aspects that their involvement could represent. They described how the adolescents were admitted and gained weight, but there was no psychological insight or motivation to maintain their weight. Nevertheless, they were discharged despite their parents’ advice. This contributed negatively to a very unstable time, and from the parents’ perspectives, many unnecessary transitions.

She [our child] said to us: When I am there, I just feel like a gigantic eating disorder. I am nothing else. She did not feel seen as a person, just a weight. And I understood her.

### Overwhelming multifaceted responsibilities

The parents were overwhelmed by the many roles and their multifaceted responsibilities during the transition period. In addition to facilitating the transition for the adolescent and adapting to their needs, they had responsibilities toward other children, their work, and their own lives, yet they had difficulty meeting their other obligations. These ancillary pressures make the transition period challenging. They describe the period as lonely and wearing. Their responsibilities for facilitating the treatment were time-consuming. They described their days as being mostly occupied with preparing food, offering meal support, following up after meals, and driving the adolescents to treatment appointments. As their adolescents often lacked the motivation or ability to administer their contact with different caring facilities, they obtained a coordination role for their adolescent. They emphasized how their adolescent still needed the same support from them as when they were underage. Meals were often followed with anxiety for the adolescent, parents, and other family members. The preparations and routines surrounding meals were adapted specifically around the patient and not for other family members. The parents expressed concern for their other children, as their adolescent with AN would often direct their strong emotions and aggression at them.

A mother’s heart bleeds when I see how she treats her sister, but what can I do?

Since their lives were adapted to helping and looking after their adolescents, they relied on flexible jobs. Some described concerns about income, as they had increased expenses due to the need to purchase different foods and treatments. As their adolescents were often in-between treatment facilities, they felt as if they had to stay home and look after them, as they tended to be physically and mentally unstable. For the parents, this situation created stress as they were reliant on others being flexible on their behalf, which created insecure living conditions. They were left with no choice but to try to be with their adolescent and provide them with the security they needed. The transition period was thereby characterized by a lot of emotions, fear, and frustration. Some described difficulties sleeping and increased mental health problems. Nevertheless, as they had no choice, they had to endure.

I just did it. Had no choice really. That is the problem. And kids are your kids until they do not need you anymore. So, we have followed her very closely, but I think this is something all parents do.

### Parents are in need of professional support

The parents described how they always had to be on guard, available, and prepared during the transition period, and they were therefore in need of support from the mental health care services. Some parents felt their adolescent had a hold on them and knew what to say to get their own way. Having experienced how life threatening their condition was and how unstable their adolescents became when setting their ground, they often submitted to their adolescents’ wishes to keep the peace. They often lacked the support they needed to stand their ground and found it difficult to set boundaries.

Because so much serious stuff has happened, I always must evaluate what is the consequence of me saying no to her. If I pay attention to myself, what could be the consequence for her? So, her consequence is always more important. There is a much greater risk for her.

They described how they stayed prepared and found it difficult to relax, as many of the parents had experienced their adolescents’ self-harm and suicide attempts. This made sleeping at night difficult and affected the whole family.

I often found her brother outside her door at night because she often ran away and went down to the railways.

They emphasized their need for support and to have somebody to talk to during the transition period. While some had an arrangement with their adolescent’s clinic, others asked for help from voluntary support services or had good friends with similar experiences. The parents described that they could not focus on their own social lives, as they had to focus on their adolescents. Therefore, some felt alone and lonely. Others explained that they preferred to be alone, as they always had to be prepared for a phone call that would demand giving their attention to their adolescent.

Some found it difficult to be open with others, as they felt that their situation was an abnormal state, so they kept to themselves or used only family as support. They describe the support offered from the mental health care services as inconsistent. They missed having connections with other parents and access to more personal contact with their adolescents’ clinicians to get more adapted advice and support. Once the transition period ended and things were more stable, they had different reactions. While some were able to regain their social life and reclaim their lives, others had more difficulty letting go and still felt the strain of the accumulated stress.

In fact, for 2 years I have struggled a bit with fatigue. I kind of thought it might have something to do with the fact that I now can relax. You have walked on your toes for years, and suddenly there is no danger anymore. You feel so heavy in a way.

## Discussion

To our knowledge, this study is the first to examine the transition process between CAMHS and AMHS, focusing on the parents’ perspective toward patients with AN. The present study revealed interacting factors important for the transition. The parents describe an abrupt discharge from AMHS based on age rather than maturity. They experienced a distance between the services that resulted in a gap in the care provided. This lack of continuity in treatment often caused negative treatment circles involving repetitive contacts with PCPs and AMHS, which led to a deterioration of the adolescent’s health. Parents also described not being involved in the transition process. They felt left with the same responsibilities as in CAMHS but had less access to information. This loss of control triggered their need to be involved, not just informed. Trusting the therapists’ knowledge was important. Combined with the responsibility they felt for their adolescent wellbeing, the parents had overwhelming multifaceted responsibilities they find difficult to balance. More parents need professional support to manage this challenging time to ease their distress.

### Abrupt, discontinued transitions make parents feel responsible for coordinating the treatment

The current study shows that the parents find it important to consider the patients’ readiness, rather than just age, to successfully transition from CAMHS to AMHS. Maturity and the ability to be self-sufficient are key patient-related factors that the parents perceive as underestimated in the treatment system. During the transition, the patients were unprepared for the difference in expectations toward them, which in some cases formed a care gap where the parents were left responsible. These findings are in line with our previous study that showed how patients felt unprepared for the expectations of self-sufficiency in the AMHS and how their parents’ role changed during the transition phase [14]. The patients in our previous study found the transition process abrupt, and they had little influence on what they described as a critical period in their treatment. The loss of a safe environment and being expected to be more autonomous than what they were prepared for increased their anxiety and ambivalence. While supporting the adolescent, the parents in the current study spent much of their time during the transition process searching for adequate treatment. As they were often turned away by the AMHS, they had to assume responsibility for the patient in collaboration with their PCP. Other studies have found that, even though therapists in CAMHS expect the patient to need further treatment in AMHS, they are not directly referred to AMHS [11].

Dimitropoulos, Tran [27] showed that professionals acknowledged the parents’ role in the transition and shared the concern of a more individual-oriented treatment that overlooked the need for more support by parents in the transition. Patients often had concerns over the parents’ decreased role in their experience, as they were essential in their recovery process [27].

Additionally, the lack of continuity between CAMHS and AMHS left the parents and patients without support from CAMHS when establishing a safe connection with therapists in AMHS. Bucci, Roberts [28] underlined how planning transitions can buffer the loss of a secure base in CAMHS, as it would provide security and stability for both the patients and parents. Hill, Wilde [15] explained how preparing for the transition is difficult by acknowledging how treatment systems are fragmented and AMHS thresholds make accessing treatment more difficult. Nevertheless, it is important to try to overcome these practical barriers. The current study supports the arguments for securing the transition by considering the individual family’s needs. An individual transition plan would help reduce stress and deterioration of illness, benefitting all those involved in the transition [29].

### Knowledge and involvement – important for developing a trusting relationship between parents and clinician’s

This study found that knowledge was an important part of the transition process and the basis of the parent’s trust in the clinician. Regarding the clinician’s knowledge about eating disorders, knowledge of the parents’ role in the transition, and the clinicians’ competence to include parents’ knowledge during the transition period. Treasure, Whitaker [30] has argued that it is a misconception that adult services must exclude parents from the assessment and treatment of adult patients with AN to protect the patients’ growing autonomy and confidentiality of the treatment. Clinicians treating patients with AN need competence to balance the patient’s need for independence against the parents’ involvement in treatment [30]. As our study reveals, it was sometimes difficult for clinicians to know when and how to involve the parents when treating patients with AN. As parents experience having the same responsibilities for the adolescent in the transition process as when they were treated in CAMHS, they feel left feeling alone with overwhelming responsibility. Adolescents transferring from CAMHS to AMHS often live with their families longer. In addition, their families tend to be involved to a larger degree in their diseased adolescents’ lives compared with healthy peers [19]. Other studies and international guidelines have underlined the importance of involving parents in the transition [3, 31]. However, this study’s results reveal that following up on these recommendations is challenging. Organizational factors, exemplified by a fragmented health-care system, and differences in treatment cultures, exemplified by a more individual approach in AMHS, can explain why the parents in our study experience are left uninvolved in treatment. Besides, according to a previous study that explored professionals’ experiences with the transition, clinicians have difficulties trusting their competence when treating patients with AN [32], which can influence how they trust their judgment concerning including parents. Hill, Wilde [15] identified that AMHS clinicians experience anxiety and lack confidence in their competence regarding developmental and adolescent issues; therefore, the transition period disclaims any training needs. This finding resonates with this study’s findings, where parents experienced inconsistencies between clinicians in terms of their approaches to the parents and their consciousness of their adolescents’ developmental stage. To ensure that the transition is effectuated with a high level of competence that includes the parent’s expertise knowledge of the adolescent, we recommend updating the guidelines for eating disorders to prevent the random way transitions occur and support clinicians in a difficult treatment period.

### Parents need professional support

Our study reveals how parents’ distress during the transition period is related to a lack of support in a period in which they have an overwhelming degree of multifaceted responsibilities. The most obvious finding that emerges from the analysis is that parents put the adolescent’s needs before much else due to their condition’s severity. This finding is expected, as other studies have reported the heavy load and disruption to caregivers’ lives due to the nature and demands of their adolescent’s illness [20, 33]. It also pinpoints how differently parents experience their social support and how open and honest they can be with close friends and family. Kyriacou, Treasure [20] argued that both patients’ and parents’ needs must be identified and addressed in the transition. Earlier studies revealed how the development of overprotective parenting styles and expressing high emotion are common reactions for parents’ caregiving for patients with AN. These reactions are associated with caregiver distress and burnout [30, 34]. Roots, Rowlands [35] found that parents experienced relief and unease when excluded from therapy, which resonates somewhat with this study’s findings. The criterion is that parents need to feel safe and trust the patient’s clinician. As in our study, Roots et al. (2009) found that parents and siblings need and value support from clinicians. We recommend that the clinicians facilitating the transition consider the important role parents have, being a secure epicenter for the adolescents in a turbulent period in their lives. Although studies have shown how social support can be a significant moderator of the stress following the caregiving role [36], we believe establishing family support within health care services could decrease the negative aspects of caregiving during the transition process.

### Limitations

The study has generated findings from qualitative interviews concerning 10 transitions. The adolescents going through the transitions were all female, and the majority of the parents were also female. As the participants were recruited through therapists who perceived the transition to be an important theme and selected from volunteers, we may have recruited a biased sample most familiar with negative consequences with the transition process. Norwegian mental health system and cultural aspects may have provided this study with themes other than what other cultures would generate. However, our findings support those found in international literature and can be assumed to have relevance in other cultural settings.

## Conclusion

Improving the transition by focusing on including parents and adolescents and preparing them for the transition period could ease parental distress and improve adolescents’ compliance with treatment. Clinicians should increase their focus on the important role of parents in the transition process. There may be a need for more extensive service changes; the system should implement routines and guidelines to offer caregivers support and guidance during the transition process.

## Data Availability

The datasets used and/or analyzed during the current study are available from the corresponding author upon reasonable request.

## Abbreviations

CAMHS: Child and Adolescent Mental Health Services
AMHS: Adult Mental Health Services
AN: Anorexia Nervosa
PCP: Primary Care Doctor
